# High-Content Analysis-Based Sensitivity Prediction and Novel Therapeutics Screening for c-Met-Addicted Glioblastoma

**DOI:** 10.3390/cancers13030372

**Published:** 2021-01-20

**Authors:** Jeong-Woo Oh, Yun Jeong Oh, Suji Han, Nam-Gu Her, Do-Hyun Nam

**Affiliations:** 1 Institute for Refractory Cancer Research, Samsung Medical Center, Seoul 06351, Korea; dhwjddn@korea.ac.kr (J.-W.O.); yunjeong.oh@sbri.co.kr (Y.J.O.); 2 Department of Health Sciences & Technology, Samsung Advanced Institute for Health Science & Technology, Sungkyunkwan University, Seoul 06351, Korea; 3 Research Institute, National Cancer Center, Goyang 10408, Korea; hanbonoboss@ncc.re.kr; 4 R&D Center, AIMEDBIO Inc., Seoul 15835, Korea; 5 Department of Neurosurgery, Samsung Medical Center, Sungkyunkwan University, Seoul 06351, Korea

**Keywords:** high-content analysis, targeted therapeutics, c-Met inhibitor, CDK4/6 inhibitor

## Abstract

**Simple Summary:**

Real-time ex vivo drug testing tailors individual therapeutics based on predicted drug responses. Most technologies to date rely on conventional drug screening that provides low confidence data. Here, we present high-content analysis-based drug testing of glioblastoma patients to identify the right glioblastoma patients for a given drug. This generates multi-parameter biomarker and phenotype readouts providing a better reliability of the assay. Additionally, we showed a high-content drug repurposing screen and defined a new c-Met-inhibiting function of the CDK4/6 inhibitor Abemaciclib. Large-scale high throughput screening results demonstrate that Abemaciclib sensitivity in glioblastoma patients is highly correlated with the c-Met inhibitors sensitivity, further supporting the accuracy of the platform and important new clinical implications regarding multiple functions of Abemaciclib.

**Abstract:**

(1) Background: Recent advances in precision oncology research rely on indicating specific genetic alterations associated with treatment sensitivity. Developing ex vivo systems to identify cancer patients who will respond to a specific drug remains important. (2) Methods: cells from 12 patients with glioblastoma were isolated, cultured, and subjected to high-content screening. Multi-parameter analyses assessed the c-Met level, cell viability, apoptosis, cell motility, and migration. A drug repurposing screen and large-scale drug sensitivity screening data across 59 cancer cell lines and patient-derived cells were obtained from 125 glioblastoma samples. (3) Results: High-content analysis of patient-derived cells provided robust and accurate drug responses to c-Met-targeted agents. Only the cells of one glioblastoma patient (PDC6) showed elevated c-Met level and high susceptibility to the c-Met inhibitors. Multi-parameter image analysis also reflected a decreased c-Met expression and reduced cell growth and motility by a c-Met-targeting antibody. In addition, a drug repurposing screen identified Abemaciclib as a distinct CDK4/6 inhibitor with a potent c-Met-inhibitory function. Consistent with this, we present large-scale drug sensitivity screening data showing that the Abemaciclib response correlates with the response to c-Met inhibitors. (4) Conclusions: Our study provides a new insight into high-content screening platforms supporting drug sensitivity prediction and novel therapeutics screening.

## 1. Introduction

Glioblastoma is the most common primary intracranial tumor, with a median survival of only 14 months [1]. Currently available treatment options for patients with glioblastoma are few other than radiation and chemotherapy [2,3]. Due to its high tumor heterogeneity and malignancy, glioblastoma relapses over time after chemoradiotherapy and is incurable [4]. Despite a huge effort in the development of new therapeutics over the last decade, few have proven effective so far [5,6,7,8]. Therefore, there are high unmet clinical needs for improving the survival of glioblastoma patients.

c-Met is a receptor tyrosine kinase (RTK) that regulates diverse cellular processes such as proliferation, survival, cell migration, and invasion [9]. c-Met act as a receptor for the ligand hepatocyte growth factor (HGF) and transduces the signal to the intracellular proteins to stimulate their biological functions. Once it is activated by phosphorylation, c-Met regulates the docking protein GAB1 and intracellular mediators such as PI3K, SHP2, GRB2, and STAT3 [10]. Aberrant HGF/c-Met axis is frequently found in several malignancies including glioblastoma, gastric, and lung cancer [11,12]. Amplification of the c-Met gene is observed in 1.6–4% of glioblastomas, and its overexpression appears more frequent [13,14]. Thus, c-Met is a well validated target for cancer therapeutics, and several c-Met-targeting drugs have been FDA (Food and Drug Administration)-approved or are being evaluated in clinical stages [15,16].

Ex vivo drug testing of patient-derived tumor cells has emerged as an important platform to guide clinical decision-making. A recent high throughput screening using a pan-cancer tumor spheroid reported the first large-scale pharmacogenetic study [17], and cancer types were expanded to gynecologic and gastric cancers [18,19]. In addition to traditional high throughput screening, high-content analysis providing cellular phenotypes and biomarkers enables more diverse aspects of drug response to be acquired [20,21].

In this study, we used a high-content analysis platform to examine c-Met-overexpressed patient-derived cells and test their response to c-Met-targeted agents. We measured c-Met immunofluorescence and multi-parameter cellular phenotypes, and identified 1 out of 12 glioblastoma patients who would be likely to respond to c-Met-targeted drugs. Genomic analysis and immunoblotting confirmed that the patient had c-Met overexpression, suggesting the reliability of the platform. Additionally, we identified CDK4/6 inhibitor Abemaciclib as an inhibitor of c-Met phosphorylation by screening the patient-derived cells with 60 different drugs. Our data provide a new insight into the possibility of high-content analysis strategies in ex vivo drug testing and precision oncology.

## 2. Materials and Methods

### 2.1. Patient-Derived Glioblastoma Tumor Cells

This study was approved by the Institutional Review Board (IRB file #201512092) of Samsung Medical Center, and informed consent was obtained from all 12 patients. Tumor specimens were resected from 12 patients who were diagnosed with glioblastoma. Patient-derived tumor tissues were dissociated using an enzyme and incubated in neurobasal-A media (Gibco, Grand Island, NY, USA) supplied with N2, B27, L-Glutamin (Gibco), penicillin streptomycin (Gibco), human recombinant basic fibroblast growth factor (bFGF), and epidermal growth factor (EGF), as previously reported [21]. Cells or frozen cells within 4 weeks after biopsy were used.

### 2.2. Cell-Lines

EBC-1 and MKN45 cells were purchased from the American Type Culture Collection (ATCC). Cells were grown in RPMI (Roswell Park Memorial Institute) with 10% fetal bovine serum and 1% antibiotics.

### 2.3. Reagents and Antibodies

c-Met inhibitors (Cabozantinib-#S1119, Crizotinib-S1068, Foretinib-#1111, Capmatinib-#S2788) and CDK4/6 inhibitors (Abemaciclib-#S7158, Palbociclib-#S1116, Ribociclib-#S7440) were purchased from Selleckchem (Houston, TX, USA). The c-Met-targeting antibody, SAIT301 was produced according to the previous literature [22]. Antibodies for anti-c-Met (#8494S) and anti-phospho-c-Met (#3077S) were purchased from Cell Signaling. Anti-β actin (#ab8227) was purchased from Thermo Fisher Scientific (Waltham, MA, USA). Laminin (#L2020) and DMSO (#D2650) were purchased from Sigma Aldrich (St. Louis, MO, USA) and normal human IgG control (#1-001-A) was purchased from R&D Systems (Minneapolis, MN, USA). Hoechest33342 (#H3570) was purchased from Life Technologies (Carlsbad, CA, USA).

### 2.4. Image-Based High-Content Screening and Analysis

Laminin was coated onto a CellCarrier 384 well plate (PerkinElmer, Waltham, MA, USA) as previously reported [23]. A total of 4000 cells were seeded in each well, treated with drugs after 24 h, fixed with 4% paraformaldehyde, and follow-up with blocking with 1% BSA(Bovine Serum Albumin) and 0.3% Triton X-100 dissolved in PBS (Phosphate-buffered saline) as previously reported [21]. Then, cells were incubated overnight at 4 °C with anti-c-Met antibody or anti-phospho-c-Met antibody, followed by incubation with Alexa Fluor 488 secondary antibody (#A-11008, Invitrogen, Carlsbad, CA, USA). Cells were stained with Hoechest33342. Cell images and real-time live cell images were acquired with Operetta CLS (PerkinElmer) and analyzed using Harmony Software (PerkinElmer) according to the manufacture’s guideline and a previous report [21].

### 2.5. Immunoblot Assay

Immunoblot assay was conducted as previously described [24]. Briefly, cell pellets were lysed with cOmplete lysis-M buffer (Roche Applied Science, Penzberg, Germany), and the supernatant was collected after centrifugation. Protein was quantified using the Bradford assay kit (Bio-Rad, Hercules, CA, USA) and mixed with laemmli SDS (Sodium Dodecyl Sulphate) sample buffer (Bio-Rad) followed by heating at 95 °C, 5 min. Then, the same amount of protein samples was loaded in SDS-PAGE (Sodium Dodecyl Sulphate–Polyacrylamide Gel Electrophoresis) and transferred to a PVDF (Polyvinylidene fluoride) membrane using the iBlot 2 system (Life Technologies). The membrane was blocked with 1% BSA for 1 h, incubated with primary antibodies overnight at 4 °C and incubated with secondary antibodies for 1 h. SuperSignal West Pico Plus (Thermo Fisher Scientific) was used to detect the protein band.

### 2.6. Cell Viability, Apoptosis Assay, and Statistics

Cells were seeded in a laminin-coated 384-well plate (2000 cells in 40 uL culture media/well); after 24 h, drugs were treated for 7 days. Cell viability was measured using either image-based live cell analysis or adenosine triphosphate (ATP)-based cell viability assay as previously described [17]. For the cell-counting analysis, the nuclei of live cells were segmented and counted. Apoptotic cells were measured with Caspase3/7 Green Detection Reagent (Invitrogen). AUC (Area Under the Curve) and IC50 (half maximal inhibitory concentration) values were analyzed by using Prism 7 software (Graphpad, San Diego, CA, USA). AUC values were obtained through the area under the points using the trapezoid rule, while IC50 values were obtained from the following analysis model: [log(inhibitor) vs. response—Variable slope] and the equation is Y = Bottom + (Top-Bottom)/(1 + 10[(LogIC50-X) * HillSlope]).

### 2.7. RNA Sequencing

Targeted exome sequencing that covers only glioma-related genes was conducted for GBM (Glioblastoma Multiform) patient-derived cells. The resulting sequence data were mapped to the human genome (hg19) with the Burrows−Wheeler Aligner (BWA) as previously described [24].

### 2.8. Kinase Assay

The in vitro kinase assay was performed using the ADP-Glo + MET Kinase Enzyme System (Promega, Madison, WI, USA) according to the manufacturer’s instructions. Small molecules (carbozantinib, crizotinib, abemaciclib, palbociclib, and ribociclib) were mixed with ATP, substrate, and MET enzyme at room temperature for 60 min. ADP-Glo™ reagent was added and incubated at room temperature for 40 min. Then, kinase detection reagent was added at room temperature for 30 min. The luminescence was measured with an Envision plate reader (PerkinElmer). Resulting data were normalized to the control and analyzed regarding the dose-response curve. The equation was Y = 100/(1 + 10[(LogIC50-X) * HillSlope]).

## 3. Results

### 3.1. Molecular and Sensitivity Testing of Glioblastoma Patient-Derived Cells to c-Met-Targeted Agents

**Figure 1:**
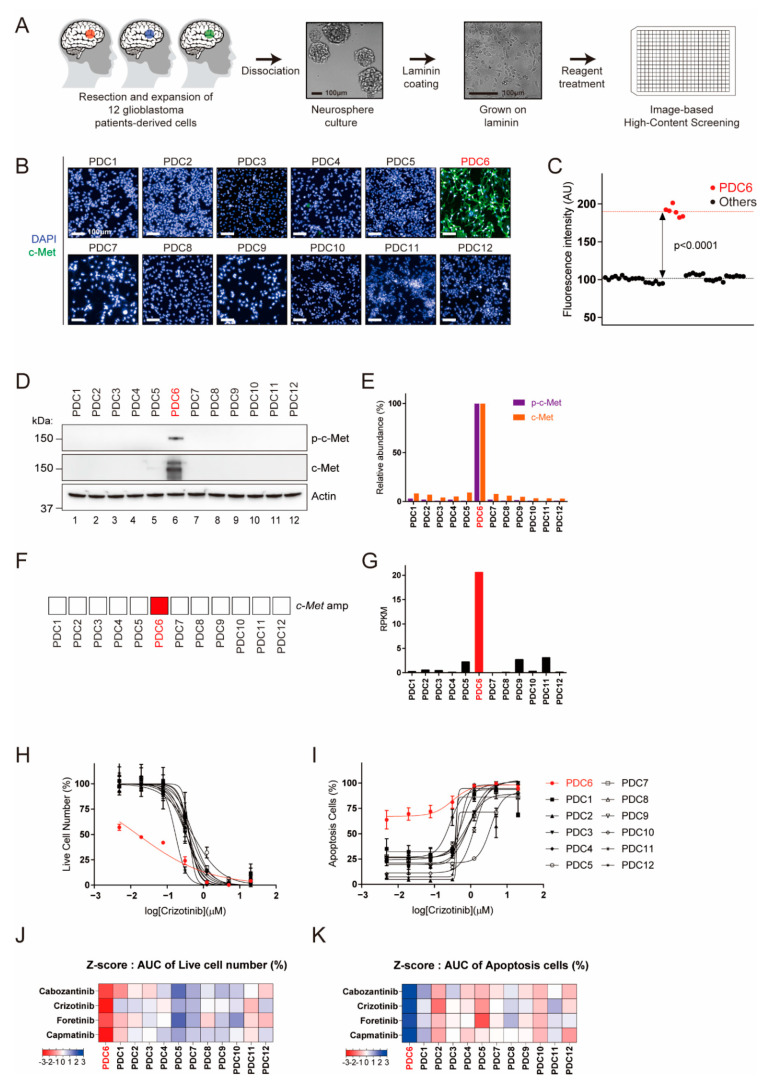
Identification of c-Met (tyrosine-protein kinase Met)-overexpressed patient-derived glioblastoma tumor cells by image-based high-content screening. (**A**) Schematic representation of glioblastoma patient-derived cell preparation and image-based high-content screening. Twelve patient-derived glioblastoma tumor specimens were resected, dissociated, and grown as neuro-sphere. The cells grown on laminin-coated plates were subjected to drug screening and immunofluorescence. (**B**) Fluorescence images of c-Met (green) and nucleus (blue) in 12 patient-derived glioblastoma cells are shown. (**C**) c-Met intensity measurements for 12 PDCs (Patient Derived Cells). PDC6 have a significantly higher c-Met level among the cells (*p* < 0.0001). (**D**) The p-c-Met and c-Met levels in 12 PDCs were examined using immunoblotting assay. Actin was used as a loading control. (**E**) The levels of p-c-Met and c-Met relative to Actin were quantified and represented in a bar graph. (**F**) Map of the c-Met gene amplification status. (**G**) Reads per kilobase of transcript per million mapped reads (RPKM) values of c-Met are shown in a bar graph. (**H**,**I**) Dose-response curve (DRC) graph of viable cell number and apoptosis cells in response to crizotinib (20 μm–4.9 nM) are shown. (**J**,**K**) Area under the curve (AUC) were analyzed from DRCs of cabozantinib, crizotinib, foretinib and capmatinib in 12 cells. AUC-based z-scores in each drug were summarized in heat maps. Color scales with z-score values are indicated.

We previously suggested that patient-derived live cells can be subjected to ex vivo drug testing to identify potentially effective drugs [17,21]. To concurrently obtain the c-Met protein level and drug sensitivity in a single platform, we developed an assay for c-Met immunofluorescence in a 384-well plate format (Figure 1A). For 12 glioblastoma cells evaluated, only one (PDC6) showed significantly high c-Met immunofluorescence (Figure 1B,C). However, it appears that the other cells had extremely low c-Met levels comparable to the background intensity. To confirm this, we conducted immunoblotting to examine the steady-state levels of c-Met and phospho-c-Met (p-c-Met). As shown in Figure 1D,E, c-Met and p-c-Met were only detected in PDC6. Genomic and RNA sequencing analysis also showed that PDC6 cells have amplification and Exon14 skipping mutation of the c-Met gene with a high RPKM (reads per kilobase of transcript per million mapped reads) (Figure 1F,G). Additionally, analysis of image-based cell viability assay showed PDC6 cells were selectively sensitive to crizotinib (Figure 1H) and other c-Met-targeted drugs, cabozantinib, foretinib and capmatinib (Figure S1A). Likewise, these c-Met-targeted drugs induced substantial apoptosis in PDC6 cells (Figure 1I and Figure S1B). Z-scores analyzed from the area under curve (AUC) values are summarized in Figure 1J,K, and these data indicate that only PDC6 cells respond to c-Met-targeted drugs. Taken together, we identified the c-Met-overexpressing glioblastoma cells and validated their drug sensitivity using a high-content analysis platform.

### 3.2. Multi-Parametric Characterization of Glioblastoma Cells to Anti-c-Met Antibody

**Figure 2:**
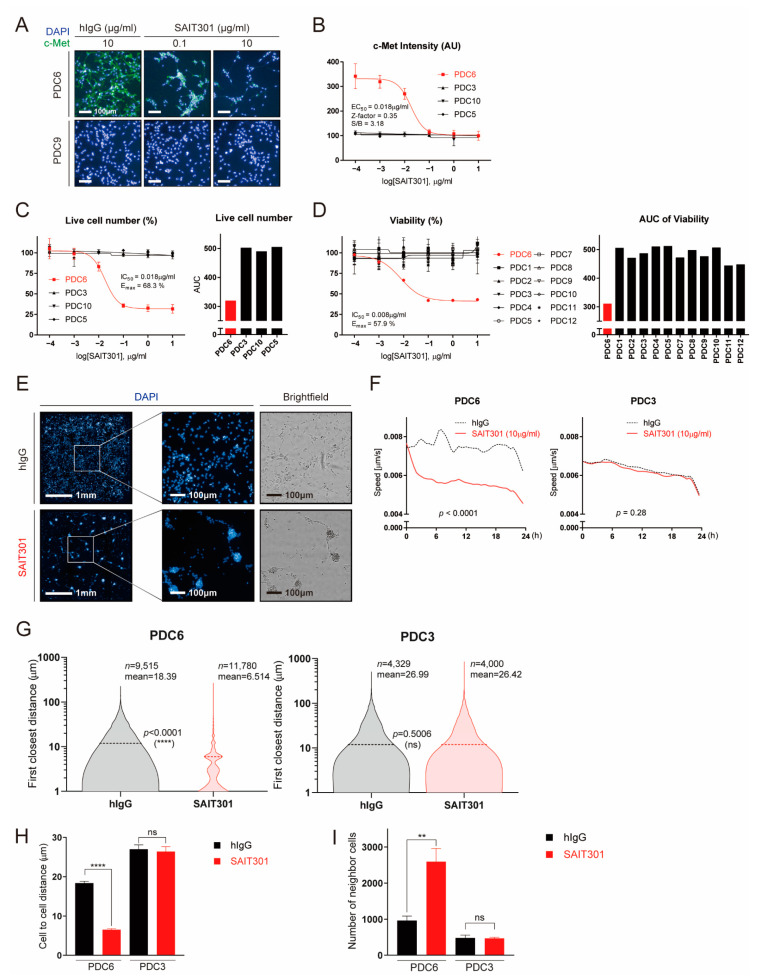
Multi-dimensional high-content analyses demonstrate the effect of SAIT301 on glioblastoma patient-derived cells. (**A**) Representative images of c-Met (green) and DAPI (4′,6-diamidino-2-phenylindole) (blue) in PDC6 and PDC9 cells treated with control IgG (10 μg/mL), SAIT301 (0.1 μg/mL, 10 μg/mL) for 24 h are shown. (**B**) DRC (Dose Response Curve) graph of c-Met immunofluorescence intensity measurements in indicated cells treated with SAIT301 (0.0001–10 μg/mL) for 24 h. (**C**,**D**) A DRC of cell viability in response to SAIT301 (0.0001–10 μg/mL) for 7 days was evaluated by either image-based high-content analysis (cell number counting, **C**) or ATP-based assay (**D**). IC50 and the maximal efficacy (Emax) are indicated in the graph. Data represent mean (*n* = 3) and SD = 1 error. AUC values are also shown on the right sides. (**E**) Representative images of DAPI (10×) and their magnified images (40×) of PDC6 cells treated with or without SAIT301 (10 μg/mL) for 24 h are shown. Brightfield images are on the right sides. (**F**) The speed of cell movement was calculated by analyzing real-time cell images taken every 30 min for 24 h. PDC3 data are shown for comparison. (**G**) PDC3 and PDC6 cells treated with either control hIgG (Human Immunoglobulin G) (10 μg/mL) or SAIT301 (10 μg/mL) were analyzed at the single-cell level. The first closest distance (pixel) of single cells is shown as violin plots. Cell numbers used in analysis are indicated with the mean value and *p*-value. (**H**) Mean value of cell-to-cell distance (μm) and (**I**) number of neighbor cells (fold change) (****: *p* < 0.0001, **: *p* < 0.01, ns: *p* > 0.05, not significant).

Next, we explored the feasibility of additional assays that can generate more readouts by high-content analysis. SAIT301 is a humanized c-Met antibody that causes degradation of the c-Met protein [22]. In response to SAIT301, the c-Met immunofluorescence drastically decreased in PDC6 cells, while c-Met level was invisible in PDC9 cells regardless of the antibody treatment (Figure 2A). SAIT301-caused c-Met protein decrease was also confirmed by immunoblot (Figure S2). The measured half-maximal c-Met inhibitory concentration (IC50) of SAIT301 in PDC6 was 18 ng/mL (Figure 2B). Cell viability analysis with increasing concentrations of SAIT301 are shown in Figure 2C. Half-maximal growth-inhibitory concentration (IC50) and maximal efficacy (Emax) of SAIT301 in PDC6 were 18 ng/mL and 68.3%, respectively. Of note, ATP-based cell viability assay resulted in IC50 of 8 ng/mL and Emax of 57.9%, validating the consistency and reliability of high-content analysis data (Figure 2D).

One of the key features of PDC6 cells upon SAIT301 treatment was cell aggregation and reduced cell motility (Figure 2E). We measured the speed of cell migration using real-time cell images taken every 30 min for 24 h. As shown in Figure 2F, SAIT301 significantly suppressed cell migration in PDC6 (*p* < 0.0001), while PDC3 cells were largely unaffected (*p* = 0.28). We also acquired the single cell level images and analyzed the closest distance between individual cells. As shown in Figure 2G, SAIT301-treated cells were highly aggregated (*n* = 11,780, mean distance: 6.514 μm) compared to human IgG control (*n* = 9515, mean distance: 18.39 μm). However, we identified no difference in cell-to-cell distances in PDC3 between IgG control (*n* = 4329, mean distance: 26.99 μm) and SAIT301 (*n* = 4000, mean distance: 26.42 μm). The mean average of cell aggregation induced by SAIT301 was statistically significant in PDC6 (*p* < 0.0001) but not in PDC3 (*p* = 0.5006) (Figure 2H). Furthermore, the number of neighbor cells were greatly increased by SAIT301 in PDC6 owing to reduced cell motility (Figure 2I). Collectively, we conclude that high-content analysis of glioblastoma patient-derived cells can efficiently provide multi-parametric drug responses including diverse molecular and phenotypic readouts.

### 3.3. Identification of Abemaciclib as an Inhibitor of c-Met

**Figure 3:**
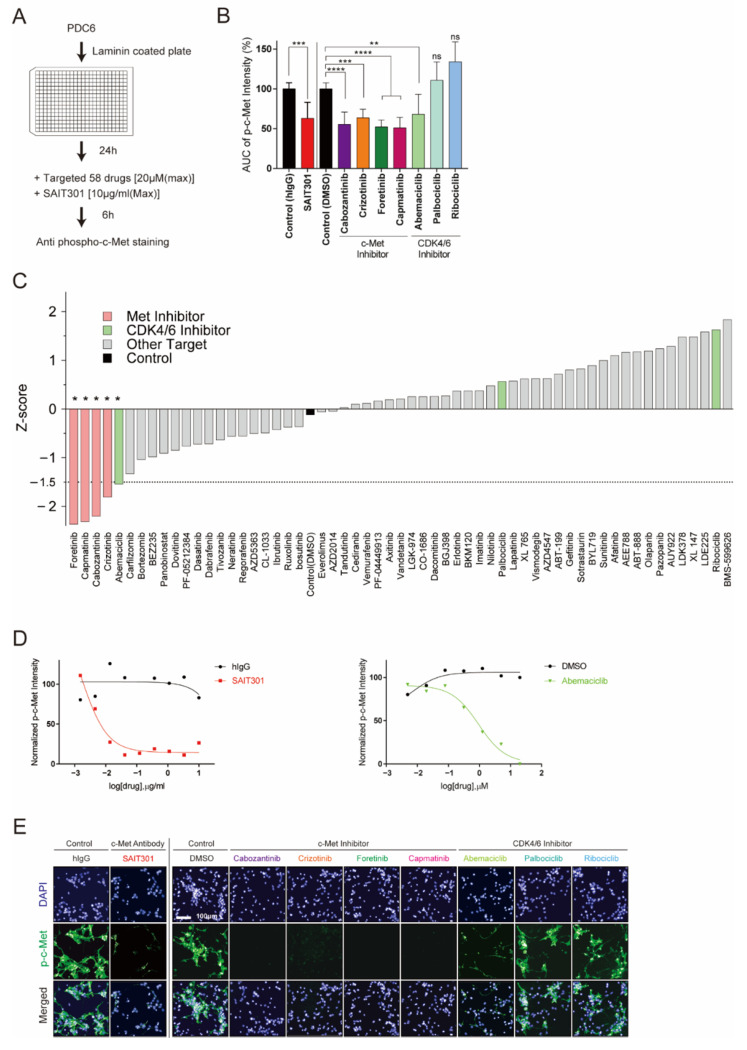
Drug repurposing screen identifies abemaciclib as an inhibitor of c-Met. (**A**) Overview of the drug repurposing screen. PDC6 were treated with 58 targeted agents and SAIT301, and image-based p-c-Met fluorescence assay was performed. (**B**) AUC was obtained from DRC of p-c-Met assay. Representative graph shows AUC of SAIT301 and four known c-Met-targeting inhibitors and three CDK4/6 inhibitors (****: *p* < 0.0001, ***: *p* < 0.001, **: *p* < 0.01, *: *p* ≤ 0.05, ns: *p* > 0.05, not significant). (**C**) Z-score of DMSO and 58 drugs for p-c-Met screen. (**D**) DRC of normalized p-c-Met intensity for SAIT301 (left) and abemaciclib (right). (**E**) Fluorescence images of DAPI (blue) and p-c-Met (green) in PDC6 treated with SAIT301(10 μg/mL), four known c-Met-targeting inhibitors (20 μm), and three CDK4/6 inhibitors (20 μm).

PDC6 cells were validated as c-Met-addicted glioblastoma cells as we characterized their genetic, molecular, and drug sensitivity information by high-content analysis and traditional methods. Next, we tested the feasibility of a drug repositioning screen using PDC6 cells and high-content analysis to explore new therapeutics that potentially inhibit c-Met. PDC6 cells were grown on laminin, treated with increasing concentrations of 58 different targeted agents, and p-c-Met intensity was analyzed (Figure 3A). Dose-response results of mean p-c-Met intensity and relative AUC value were analyzed. As expected, all c-Met-targeted drugs such as crizotinib, cabozantinib, foretinib, capmatinib, and SAIT301 were shown to markedly decrease the p-c-Met level (Figure 3B, Table 1). Intriguingly, one CDK4/6 inhibitor known as abemaciclib was found to suppress p-c-Met intensity. Since palbociclib and ribociclib did not affect the p-c-Met level, c-Met regulation is not likely to be a general mechanism of CDK4/6 inhibition (Figure 3B). All drugs tested were ranked by z-score and are shown in Figure 3C. Drugs with lower z-score than the threshold of −1.5 were considered potentially effective. Besides known c-Met-targeted agents, only abemaciclib met this criteria (z-score: −1.54), while no significant change was observed in palbociclib (z-score: 0.57) and ribociclib (z-score: 1.63) (Figure S3). Dose-response results testing SAIT301 (Figure 3D, left, Table 2) and abemaciclib (Figure 3D, right, Table 2) in PDC6 cells showed concentration-dependent changes in the p-c-Met level. We also sought to check high-content screen images to validate quantified immunofluorescence data. As shown in Figure 3E, all validated c-Met-targeted drugs and abemaciclib led to clear decrease in p-c-Met intensity. These data suggest that abemaciclib had a mechanism distinct from other CDK4/6 inhibitors regulating c-Met.

### 3.4. Unique Role of CDK4/6 Inhibitor Abemaciclib in c-Met Regulation

**Figure 4:**
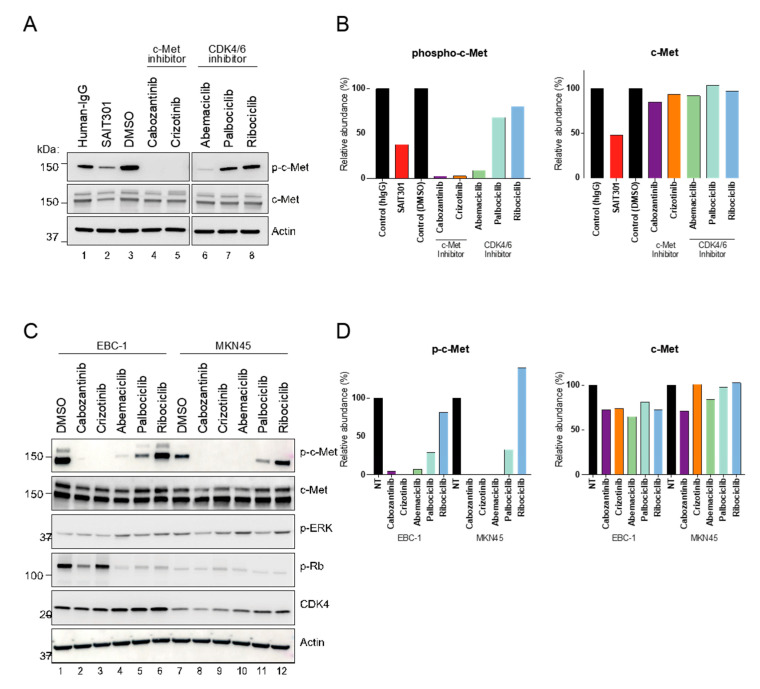
Distinct role of abemaciclib in regulation of c-Met activity. (**A**) PDC6 were treated with 10 μm of cabozantinib, crizotinib, abemaciclib, palbociclib, and ribociclib, and 100 nM of SAIT301 for 12 h. p-c-Met and c-Met levels were examined by immunoblotting. Human IgG and Actin were used as a loading control. (**B**) Steady state levels of p-c-Met relative to Actin and c-Met relative to Actin. (**C**) EBC1 and MKN45 cells were treated with 10 μm of cabozantinib, crizotinib, abemaciclib, palbociclib and ribociclib for 12 h. p-c-Met and c-Met levels were examined by immunoblotting. Actin was used as a loading control. (**D**) Steady state levels of p-c-Met and c-Met relative to Actin, respectively.

To support our findings, we performed immunoblot on PDC6 cell extracts treated with the drugs. Abemaciclib led to a dramatic decrease in the p-c-Met level in PDC6 cells, while palbociclib and ribociclib had no effect (Figure 4A,B). Total c-Met protein was not affected by abemaciclib, suggesting the mechanism by which abemaciclib inhibits p-c-Met is not related to steady-state abundance of total c-Met protein. To further address if these are cell type-specific effects, we conducted immunoblot on extracts from c-Met-overexpressing cancer cell lines EBC-1 (lung) and MKN45 (gastric) [25]. Consistent with the glioblastoma cell results, abemaciclib remarkably inhibited the p-c-Met level in both cancer cell lines (Figure 4C,D). It is notable that palbociclib had a modest effect in both cell lines, whereas ribociclib had no effect. All CDK4/6 inhibitors showed a similar extent of phosphor-RB inhibition (Figure S4). Next, to assess whether abemaciclib acts as a direct kinase inhibitor of c-Met, we performed a biochemical kinase assay. Although cabozantinib and crizotinib showed a markedly greater effect, abemaciclib was shown to inhibit kinase activity of c-Met at high concentrations, suggesting that abemaciclib can directly inhibit the enzyme function of c-Met (Figure S5). Notably, a recent report also indicated that abemaciclib inhibits kinases other than CDK4/6 [26]. Further studies will be necessary to confirm our observations.

### 3.5. Large-Scale Drug Screening Data Suggest Sensitivity Correlation of Abemaciclib and c-Met-Targeted Drugs

**Figure 5:**
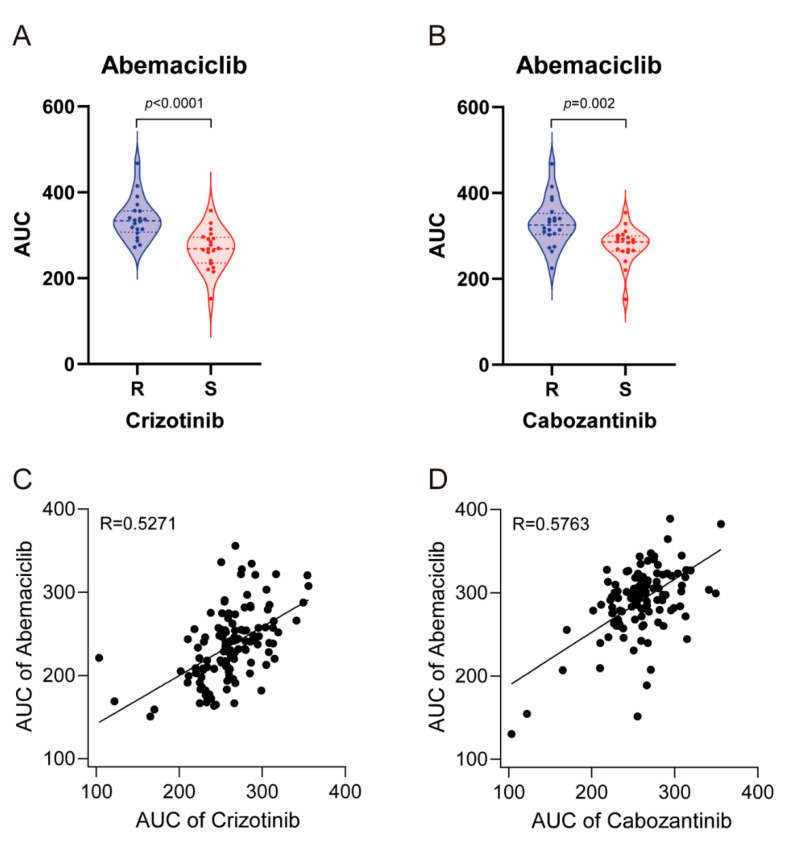
Large-scale sensitivity screening of cancer cell lines and glioblastoma patient-derived cells in abemaciclib, crizotinib and cabozantinib. (**A**) Violin plots showing AUC values of abemaciclib in 20 sensitive and 20 resistant cancer cell lines to crizotinib; *p* value is indicated in the graph. (**B**) Violin plots showing AUC values of abemaciclib in 20 sensitive and 20 resistant cancer cell lines to cabozantinib; *p* value is indicated in the graph. (**C**) Scatter plots showing AUC values of abemaciclib and crizotinib in 125 cases of patient-derived glioblastoma. Pearson’s coefficient (r) is indicated in the graph. (**D**) Scatter plots showing AUC values of abemaciclib and cabozantinib in 125 cases of patient-derived glioblastoma. Pearson’s coefficient (r) is indicated in the graph.

To investigate the physiological relevance, we compared the drug sensitivity profile of abemaciclib, crizotinib, and cabozantinib using cancer cell line screening data and previously reported large-scale pharmacogenomic study data [17]. We first screened 59 cancer cell lines with these drugs and classified them into 20 sensitive and 20 resistant cell lines to crizotinib and cabozantinib. As shown in Figure 5A,B, crizotinib-sensitive and cabozantinib sensitive cell lines were more sensitive to abemaciclib (*p* < 0.0001 and *p* = 0.002, respectively). In 125 glioblastoma patient-derived cells evaluated, we found that the sensitivity of these cells to abemaciclib was highly correlated with crizotinib and cabozantinib (r = 5271 and r = 5763, respectively) (Figure 5C,D). Taken together, these data indicate that sensitivity to abemaciclib was correlated with sensitivity to crizotinib and cabozantinib, further supporting its distinct effect on c-Met regulation.

## 4. Discussion

Precision oncology refers to the tailoring individual therapeutics based on predicted drug responses, seeking the most effective treatment for a given patient [27]. Large-scale genome sequencing studies have contributed to defining the unique genetic characteristics of human cancers [28,29,30]. Epigenetic information of a tumor can also provide prognostic and predictive information [31], and proteomics revealed that protein levels and post-translational modifications are important to understand the complexity of tumors [32,33]. In vitro drug screening efforts using human cell line models identified differential drug sensitivity in such models [34,35]. More recently, patient-derived tumor cell screening was used in the first large-scale pharmacogenetic study to guide treatment decisions [17], and high-content analysis examining cellular phenotypes and biomarkers suggested new approaches that could inform therapeutic stratification [20,21].

In this study, we present a high-content analysis-based ex vivo drug testing platform that can identify glioblastoma patients who may respond to c-Met-targeted agents. Among 12 glioblastoma patient-derived cells, only one sample (PDC6) exhibited a markedly high level of c-Met intensity with increased sensitivity to c-Met-targeted drugs. Genomic analyses validated the c-Met overexpression in PDC6 through identifying the c-Met gene amplification and mutation. Moreover, immunoblot assay detected the c-Met protein only in PDC6 cells. Since it appears that the c-Met is not detected in most glioblastoma cells, only a small subset was highly selective to c-Met-targeted agents. Therefore, the significance of selecting potential responders is high particularly in the case of c-Met-addicted glioblastoma.

Conventional in vitro drug sensitivity results alone are insufficient to understand the complex context of tumor cells owing to the absence of genetic or molecular information. High-content analysis described here offers a number of advantages over traditional high throughput screening. First, it addresses whether specific biomarkers important for drug response are significantly high or low. These provide high reliability of drug effects by explaining the target-drug association. Secondly, high-content analysis simultaneously acquires multi-parameter phenotypes. In the present study, we assessed target expression, cell viability, apoptosis, cell-to-cell distances, and real-time cell movement speed in a single experiment. This technology allows for more reliable experimental assays through concurrent collection of multi-layered data. Thirdly, high-content analysis supports the drug repositioning screen to find novel therapeutics for specific functions, which can be further exploited to reveal the underlying mode of action of the alternative mechanism of the drug. We devised a screening method using PDC6 cells to identify potential c-Met inhibiting compounds; this is not achieved with traditional high throughput screening. To inhibit the c-Met in different ways, devising a method to screen anti-WNT (Wingless-related integration site) pathway drugs in PDC6 will also be important, as the WNT pathway is a potential downstream of c-Met [36].

Abemaciclib is the most potent and the latest FDA-approved CDK4/6 inhibitor to date. Abemaciclib has a unique function with a wider range of kinase inhibition activity, suggesting it has additional mechanisms besides CDK4/6 inhibition [26]. Our findings also support this idea, since abemaciclib was identified as a potent inhibitor of c-Met through a drug repositioning screening. The biochemical kinase assay showed a high concentration of abemaciclib directly inhibited c-Met kinase activity. As abemaciclib has been shown to the penetrate blood brain barrier (BBB) and to have a favorable toxicity profile [37], expanding clinical indications such as glioblastoma possibly remains highly significant. Our data suggest that abemaciclib will be more effective in glioblastoma with the coexistence of CDK4/6 and c-Met alterations due to its dual functions. Large-scale sensitivity screening of cancer cell lines and glioblastoma patient-derived cells further provided compelling evidence that Abemaciclib sensitivity highly correlates with the c-Met inhibitor crizotinib and cabozantinib.

Collectively, our findings provide unprecedented insights into high-content analysis of patient specimens, which will contribute to empowering precision oncology and new drug discoveries. These results raise an important new possibility for high-content analysis application in a diverse area of biomarker research, drug discovery, and clinical study. Our work will facilitate expanded development of specific experimental assays and improved clinical decision-making platforms.

## Figures and Tables

**Table 1:** Statistical value of AUC;measured intensity of c-Met protein, treating c-Met inhibitors and CDK4/6 inhibitors.

Target	Drug Name	Diff. (%)	*p*-Value	Summary ^1^
c-Met Ab	SAIT301	−37	0.0004	***
c-Met Inhibitor	Carbozantinib	−44.6	<0.0001	****
	Crizotinib	−36.3	0.0007	***
	Fretinib	−47.7	<0.0001	****
	Capmatinib	−49	<0.0001	****
CDK4/6 Inhibitor	Abemaciclib	−31.9	0.0035	**
	Palbociclib	10.7	0.7588	ns ^2^
	Ribociclib	34.1	0.0016	ns

^1^ 0.01 ≥ ** > 0.001 ≥ *** > 0.0001 ≥ ****. ^2^ ns; not significant.

**Table 2:** Z’ factor of phospho-c-Met intensity between high and low concentration in each inhibitor.

Target	Drug Name	Z’ factor	Summary ^1^
c-Met Ab	SAIT301	0.35	*
c-Met Inhibitor	Carbozantinib	0.97	**
	Crizotinib	0.67	**
	Fretinib	0.75	**
	Capmatinib	0.12	*
CDK4/6 Inhibitor	Abemaciclib	0.59	**
	Palbociclib	−0.34	ns ^2^
	Ribociclib	−2.54	ns

^1^ 1 ≥ ** > 0.5 ≥ * >0 ≥ ns. ^2^ ns; not significant.

## Data Availability

The data presented in this study are available in this article (and supplementary material).

## Supplementary Materials

The following are available online at https://www.mdpi.com/2072-6694/13/3/372/s1, Figure S1: Dose response curve in 12 patient-derived samples treating c-Met target inhibitor, Figure S2: Inhibition of c-Met level treating c-Met-targeting antibody, Figure S3: CDK4/6 inhibitor abemaciclib is relatively sensitive in a Met overexpression sample like c-Met targeting small molecules, Figure S4: Dose response curve of phosphor-c-Met intensity treating CDK4/6 inhibitors and c-Met targeting inhibitors, Figure S5: Kinase assay in cabozantinib, crizotinib (c-Met inhibitor), abemaciclib, palbociclib, and ribociclib (CDK4/6 inhibitor).

## References

[1] Khosla D. (2016). Concurrent therapy to enhance radiotherapeutic outcomes in glioblastoma. Ann Transl. Med..

[2] Stupp R., Mason W.P., van den Bent M.J., Weller M., Fisher B., Taphoorn M.J., Belanger K., Brandes A.A., Marosi C., Bogdahn U. (2005). Radiotherapy plus concomitant and adjuvant temozolomide for glioblastoma. N. Engl. J. Med..

[3] Gallego O. (2015). Nonsurgical treatment of recurrent glioblastoma. Curr. Oncol..

[4] Stupp R., Hegi M.E., Mason W.P., van den Bent M.J., Taphoorn M.J., Janzer R.C., Ludwin S.K., Allgeier A., Fisher B., Belanger K. (2009). Effects of radiotherapy with concomitant and adjuvant temozolomide versus radiotherapy alone on survival in glioblastoma in a randomised phase III study: 5-year analysis of the EORTC-NCIC trial. Lancet Oncol..

[5] Brown C.E., Alizadeh D., Starr R., Weng L., Wagner J.R., Naranjo A., Ostberg J.R., Blanchard M.S., Kilpatrick J., Simpson J. (2016). Regression of Glioblastoma after Chimeric Antigen Receptor T-Cell Therapy. N. Engl. J. Med..

[6] Herrlinger U., Tzaridis T., Mack F., Steinbach J.P., Schlegel U., Sabel M., Hau P., Kortmann R.D., Krex D., Grauer O. (2019). Lomustine-temozolomide combination therapy versus standard temozolomide therapy in patients with newly diagnosed glioblastoma with methylated MGMT promoter (CeTeG/NOA-09): A randomised, open-label, phase 3 trial. Lancet.

[7] Stupp R., Taillibert S., Kanner A.A., Kesari S., Steinberg D.M., Toms S.A., Taylor L.P., Lieberman F., Silvani A., Fink K.L. (2015). Maintenance Therapy With Tumor-Treating Fields Plus Temozolomide vs Temozolomide Alone for Glioblastoma: A Randomized Clinical Trial. JAMA.

[8] Keskin D.B., Anandappa A.J., Sun J., Tirosh I., Mathewson N.D., Li S., Oliveira G., Giobbie-Hurder A., Felt K., Gjini E. (2019). Neoantigen vaccine generates intratumoral T cell responses in phase Ib glioblastoma trial. Nature.

[9] Trusolino L., Bertotti A., Comoglio P.M. (2010). MET signalling: Principles and functions in development, organ regeneration and cancer. Nat. Rev. Mol. Cell Biol..

[10] Viticchie G., Muller P.A.J. (2015). c-Met and Other Cell Surface Molecules: Interaction, Activation and Functional Consequences. Biomedicines.

[11] Liu X., Newton R.C., Scherle P.A. (2010). Developing c-MET pathway inhibitors for cancer therapy: Progress and challenges. Trends Mol. Med..

[12] Abounader R., Laterra J. (2005). Scatter factor/hepatocyte growth factor in brain tumor growth and angiogenesis. Neuro. Oncol..

[13] The Cancer Genome Atlas Research Network (2008). Comprehensive genomic characterization defines human glioblastoma genes and core pathways. Nature.

[14] Brennan C.W., Verhaak R.G., McKenna A., Campos B., Noushmehr H., Salama S.R., Zheng S., Chakravarty D., Sanborn J.Z., Berman S.H. (2013). The somatic genomic landscape of glioblastoma. Cell.

[15] Kazandjian D., Blumenthal G.M., Chen H.Y., He K., Patel M., Justice R., Keegan P., Pazdur R. (2014). FDA approval summary: Crizotinib for the treatment of metastatic non-small cell lung cancer with anaplastic lymphoma kinase rearrangements. Oncologist.

[16] Dhillon S. (2020). Capmatinib: First Approval. Drugs.

[17] Lee J.K., Liu Z., Sa J.K., Shin S., Wang J., Bordyuh M., Cho H.J., Elliott O., Chu T., Choi S.W. (2018). Pharmacogenomic landscape of patient-derived tumor cells informs precision oncology therapy. Nat. Genet..

[18] Sa J.K., Hwang J.R., Cho Y.J., Ryu J.Y., Choi J.J., Jeong S.Y., Kim J., Kim M.S., Paik E.S., Lee Y.Y. (2019). Pharmacogenomic analysis of patient-derived tumor cells in gynecologic cancers. Genome. Biol..

[19] Sa J.K., Hong J.Y., Lee I.K., Kim J.S., Sim M.H., Kim H.J., An J.Y., Sohn T.S., Lee J.H., Bae J.M. (2020). Comprehensive pharmacogenomic characterization of gastric cancer. Genome. Med..

[20] Chia S., Low J.L., Zhang X., Kwang X.L., Chong F.T., Sharma A., Bertrand D., Toh S.Y., Leong H.S., Thangavelu M.T. (2017). Phenotype-driven precision oncology as a guide for clinical decisions one patient at a time. Nat. Commun..

[21] Her N.G., Oh J.W., Oh Y.J., Han S., Cho H.J., Lee Y., Ryu G.H., Nam D.H. (2018). Potent effect of the MDM2 inhibitor AMG232 on suppression of glioblastoma stem cells. Cell Death Dis..

[22] Lee J.M., Kim B., Lee S.B., Jeong Y., Oh Y.M., Song Y.J., Jung S., Choi J., Lee S., Cheong K.H. (2014). Cbl-independent degradation of Met: Ways to avoid agonism of bivalent Met-targeting antibody. Oncogene.

[23] Pollard S.M., Yoshikawa K., Clarke I.D., Danovi D., Stricker S., Russell R., Bayani J., Head R., Lee M., Bernstein M. (2009). Glioma stem cell lines expanded in adherent culture have tumor-specific phenotypes and are suitable for chemical and genetic screens. Cell Stem Cell.

[24] Han S., Shin H., Oh J.W., Oh Y.J., Her N.G., Nam D.H. (2019). The Protein Neddylation Inhibitor MLN4924 Suppresses Patient-Derived Glioblastoma Cells via Inhibition of ERK and AKT Signaling. Cancers.

[25] Lutterbach B., Zeng Q., Davis L.J., Hatch H., Hang G., Kohl N.E., Gibbs J.B., Pan B.S. (2007). Lung cancer cell lines harboring MET gene amplification are dependent on Met for growth and survival. Cancer Res..

[26] Hafner M., Mills C.E., Subramanian K., Chen C., Chung M., Boswell S.A., Everley R.A., Liu C., Walmsley C.S., Juric D. (2019). Multiomics Profiling Establishes the Polypharmacology of FDA-Approved CDK4/6 Inhibitors and the Potential for Differential Clinical Activity. Cell Chem. Biol..

[27] Chae Y.K., Pan A.P., Davis A.A., Patel S.P., Carneiro B.A., Kurzrock R., Giles F.J. (2017). Path toward Precision Oncology: Review of Targeted Therapy Studies and Tools to Aid in Defining "Actionability" of a Molecular Lesion and Patient Management Support. Mol. Cancer Ther..

[28] Lawrence M.S., Stojanov P., Mermel C.H., Robinson J.T., Garraway L.A., Golub T.R., Meyerson M., Gabriel S.B., Lander E.S., Getz G. (2014). Discovery and saturation analysis of cancer genes across 21 tumour types. Nature.

[29] Hoadley K.A., Yau C., Hinoue T., Wolf D.M., Lazar A.J., Drill E., Shen R., Taylor A.M., Cherniack A.D., Thorsson V. (2018). Cell-of-Origin Patterns Dominate the Molecular Classification of 10,000 Tumors from 33 Types of Cancer. Cell.

[30] Dancey J.E., Bedard P.L., Onetto N., Hudson T.J. (2012). The genetic basis for cancer treatment decisions. Cell.

[31] McMahon K.W., Karunasena E., Ahuja N. (2017). The Roles of DNA Methylation in the Stages of Cancer. Cancer J..

[32] Pierobon M., Wulfkuhle J., Liotta L.A., Petricoin Iii E.F. (2019). Utilization of Proteomic Technologies for Precision Oncology Applications. Cancer Treat. Res..

[33] Rodriguez H., Pennington S.R. (2018). Revolutionizing Precision Oncology through Collaborative Proteogenomics and Data Sharing. Cell.

[34] Garnett M.J., Edelman E.J., Heidorn S.J., Greenman C.D., Dastur A., Lau K.W., Greninger P., Thompson I.R., Luo X., Soares J. (2012). Systematic identification of genomic markers of drug sensitivity in cancer cells. Nature.

[35] Barretina J., Caponigro G., Stransky N., Venkatesan K., Margolin A.A., Kim S., Wilson C.J., Lehar J., Kryukov G.V., Sonkin D. (2012). The Cancer Cell Line Encyclopedia enables predictive modelling of anticancer drug sensitivity. Nature.

[36] Kim K.H., Seol H.J., Kim E.H., Rheey J., Jin H.J., Lee Y., Joo K.M., Lee J., Nam D.H. (2013). Wnt/beta-catenin signaling is a key downstream mediator of MET signaling in glioblastoma stem cells. Neuro. Oncol..

[37] Xu Z., Li H., Dong Y., Cheng P., Luo F., Fu S., Gao M., Kong L., Che N. (2020). Incidence and PD-L1 Expression of MET 14 Skipping in Chinese Population: A Non-Selective NSCLC Cohort Study Using RNA-Based Sequencing. Onco. Targets Ther..

