# Supplementary material for: High-Content Analysis-Based Sensitivity Prediction and Novel Therapeutics Screening for c-Met-Addicted Glioblastoma
Source: Cancers (Basel). 2021 Jan 20;13(3):372. doi: 10.3390/cancers13030372 (PMC7864197; doi:10.3390/cancers13030372)
Supplement: Supplementary file 1 [file cancers-13-00372-s001.pdf]

Supplementary Materials:

High-Content Analysis-Based Sensitivity Prediction and Novel Therapeutics Screening for c-Met-Addicted Glioblastoma

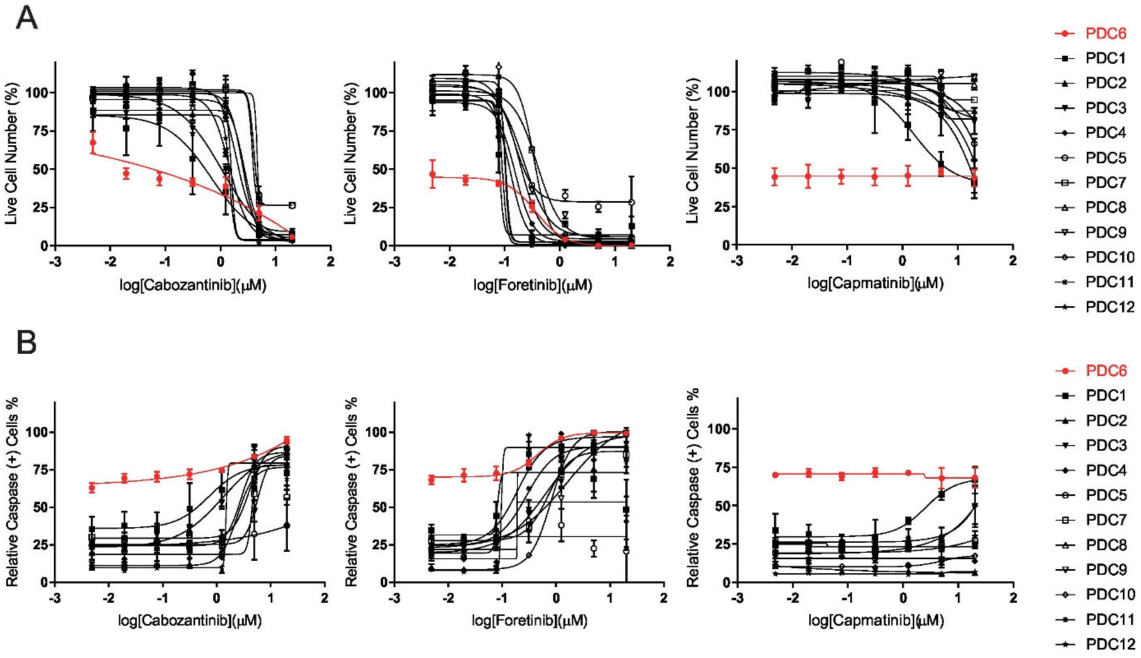

Figure S1. Dose response curve in 12 patient derived samples treating c-Met target inhibitor. (**A**) Dose-response curve (DRC) graph of cell viability in 12 glioblastoma patient-derived cells to Cabozantinib, Foretinib, Capmatinib (20μM-4.9nM) are shown. Red lines represent PDC6 cells. (**B**) DRC graph of caspase 3/7 positive cells (%) in 12 glioblastoma patient-derived cells to Cabozantinib, Foretinib, Capmatinib (20μM-4.9nM) are shown.

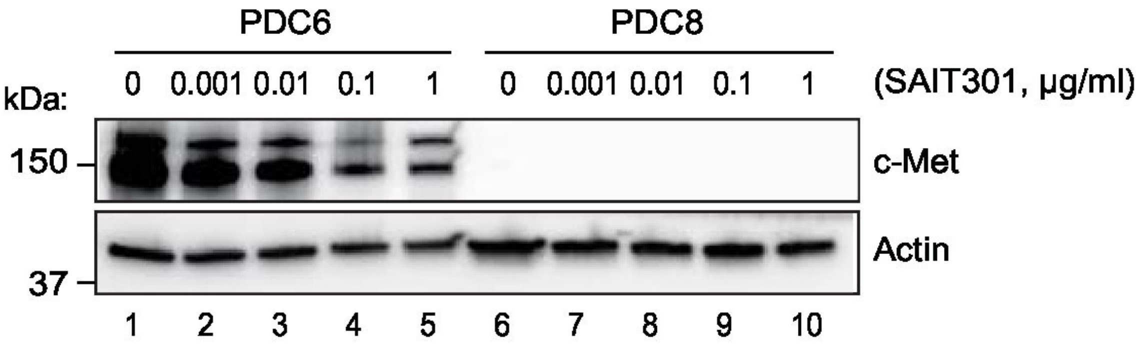

Figure S2. Inhibition of c-Met level treating c-Met targeting antibody. The c-Met levels in PDC6 and PDC8 cells were examined by immunoblotting assay. The cells were treated with 1, 0.1, 0.01, 0.001, 0μg/ml SAIT301. Actin was used as a loading control.

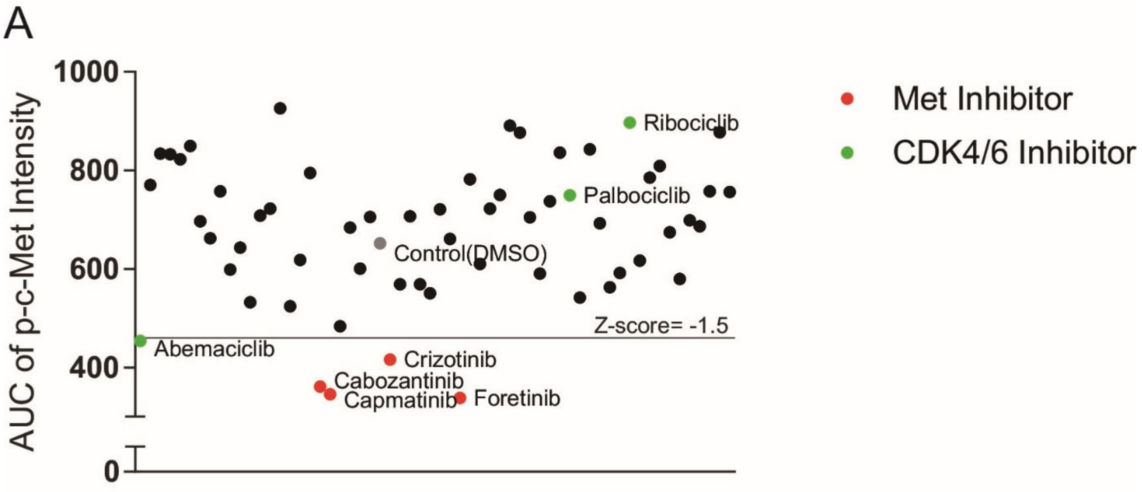

Figure S3. CDK4/6 inhibitor Abemaciclib is relatively sensitive in c-Met overexpression sample like met targeting small molecules. Representative graph shows AUC of PDC6 treated with p-c-Met intensity of 58drugs and negative control (DMSO, gray dot labeled, AUC=651.9). Z-score of -1.5 represents the threshold of statistical significance. Met inhibitors (red dot labeled) and CDK4/6 inhibitors (green dot labeled) are highlighted.

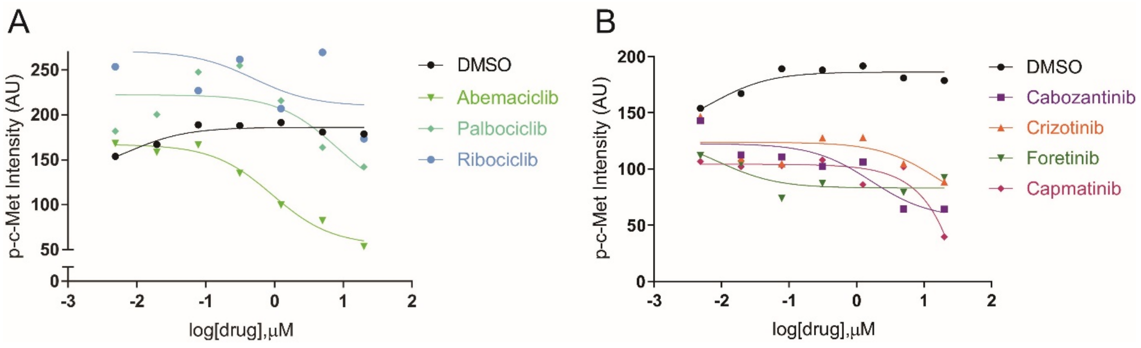

Figure S4. Dose response curve of phosphor-c-Met intensity treating CDK4/6 inhibitors and Met targeting inhibitors. (**A**) Dose-response curve (DRC) graph of p-c-Met intensity of PDC6 treated with DMSO (control) and CDK4/6 target drugs. (**B**) DRC graph of p-c-Met intensity of PDC6 treated with DMSO (control) and CDK4/6 target drugs.

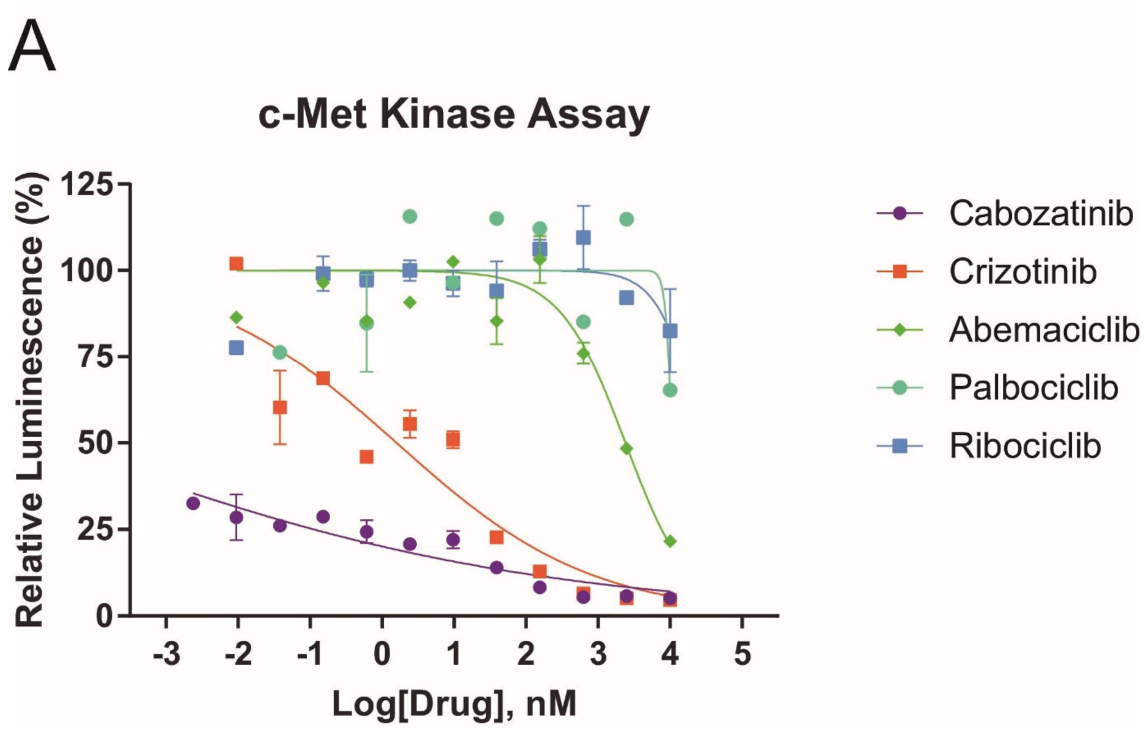

Figure S5. Kinase assay in Cabozantinib, Crizotinib(Met inhibitor), Abemaciclib, Palbociclib, Ribociclib (CDK4/6 inhibitor). (**A**) Dose-response curve (DRC) graph of kinase inhibition activity of c-Met inhibitors and CDK4/6 inhibitors.
